# Dynamics of post fire plant community assembly in Doñana coastal dunes

**DOI:** 10.1038/s41598-025-04400-x

**Published:** 2025-06-06

**Authors:** Sergio Chozas, André F. Mira, Manuel Serrano, Nagore G. Medina, Joaquín Hortal, María Cruz Díaz-Barradas

**Affiliations:** 1 CE3C - Centre for Ecology, Evolution and Environmental Changes and CHANGE - Global Change and Sustainability Institute, Faculdade de Ciências, Universidade de Lisboa, Lisbon, Portugal; 2 Department of Biogeography and Global Change, Museo Nacional de Ciencias Naturales (MNCN-CSIC), Madrid, Spain; 3 Departamento de Biología de la Conservación y Cambio Global, Estación Biológica de Doñana (EBD), Sevilla, Spain; 4 Departamento de Biología, Facultad de Ciencias, Universidad Autónoma de Madrid, Madrid, Spain; 5 Centro de Investigación en Biodiversidad y Cambio Global, Universidad Autónoma de Madrid, Madrid, Spain; 6 Departamento de Biología Vegetal y Ecología, Universidad de Sevilla, Sevilla, Spain

**Keywords:** Filtering processes, Mediterranean scrub, Modularity, Plant traits, Secondary succession, Species co-occurrence, Ecology, Plant sciences

## Abstract

Fire significantly contributes to the distribution, composition, and functioning of ecosystems. It is also among the most damaging disturbances, exacerbated by increasing fire intensity and extent due to climate change. However, some ecosystems, like Mediterranean scrublands, recover quickly due to species’ adaptations to frequent fire regimes. This study investigates the community assembly processes during a secondary succession following the fire in a dune system within Doñana Natural Park, Southern Spain. To achieve this, three shrub communities were characterised along a coast-to-inland gradient over two consecutive years using a Non-Metric Multidimensional Scaling ordination. Then, a set of ecophysiological and structural traits of the dominant shrub species were analysed through a Principal Component Analysis, and correlation analyses were performed to assess the relationships between the three communities and the studied traits. Finally, to investigate the spatial structure of the shrub communities, a co-occurrence network was constructed and Moran’s I analyses were conducted. The results revealed that natural secondary succession has restored shrub communities along the gradient to states similar to those existing before the fire, indicating a consistent recovery pattern. Although species composition was comparable across zones, community spatial structure and several traits varied from coastal to interior areas, with more complex co-occurrence networks observed inland. Overall, the results underline the significance of environmental filters and species interactions in shaping community assembly during secondary succession after fire. Furthermore, they highlight that post fire community responses can be predicted through mechanisms linking the regional species pool, regeneration traits, and physical heterogeneity.

**Supplementary Information:**

The online version contains supplementary material available at 10.1038/s41598-025-04400-x.

## Introduction

Fire plays a key role in the distribution, composition and functioning of ecosystems, regulating individual traits, community dynamics, and carbon and nutrient cycling^1,2^. It can, however, disrupt a variety of dynamic processes within an ecosystem, making it one of the most aggressive and damaging disturbance agents affecting natural habitats^3,4^. The intensity and extent of fires have been increasing with climate change, which could profoundly affect ecosystems^5,6^. Therefore, it is essential to study the impact of fire on natural communities in order to develop strategies to mitigate its impact and minimize its consequences.

After a fire event, contrasting changes in vegetation structure and diversity occur^7^, including changes in native species abundance and both positive and negative effects on species richness related to exotic species invasions or colonization of species from the landscape pool^8^. However, the early development of plant communities after fire can be accelerated by the availability of open space and soil nutrients, and low levels of biotic stress, which typically result from low plant densities^9,10^. Such mixed evidence has led to the proposal that secondary succession and community assembly after fire involve both stochastic and deterministic processes in which predictable structural and functional patterns often emerge from “residuals”, that is, individuals and propagules remaining from the pre-fire period in a sense described by MacMahon^11^, associated to the eventual arrival of new species. These patterns give rise to a range of potential successional trajectories along with varying recovery rates depending on the composition of pre-fire communities and the colonization processes^12,13^.

Some ecosystems are particularly resilient to fire. They recover easily after this disturbance, showing similar pre- and post fire structure, composition, and functioning^14^; (cf^15^. In particular, Mediterranean scrub recovers quickly after fire because its species are highly adapted to frequent fire regimes^4,10^. These species, known as pyrophytes, enable this ecosystem to return to a very similar pre-fire stage after a short period of time^16^. This involves a rapid re-establishment of biotic relationships that strongly influence community assembly and post fire succession^17,18^. For decades, ecologists have been particularly interested in exploring post fire dynamics, which are shaped by multiple factors influencing community assembly^19,20^. Abiotic factors such as solar radiation, nutrient concentration and elevated air temperature affect the establishment of the plant species at the beginning of the succession^21,22^, while biotic factors such as predation, competition, parasitism, facilitation, or mutualism become more important as the communities are progressively more structured^23^. Such interplay of factors can result in different outcomes during the filtering from the pool of plant species that can persist during succession, leading to the formation of different communities^9,24,25^. As the new communities progress and establish, they go through different phases of complexity, starting from a pioneer phase right after the fire-disturbance, followed by a building phase and eventually reaching a mature state^13^.

Coastal sand dunes are ecosystems of high conservation value located at the interface between land and sea^26^. In these environments, the natural coast-to-inland gradient of multiple environmental factors creates a typical vegetation zonation^27,28^, which provides specialized habitats for many organisms, many of those rare or endangered^29^. This gradient also makes coastal dune ecosystems ideal for studies of community assembly dynamics^30^.

The variation of species’ functional traits across environmental gradients shapes community assembly processes^31^. Differences in functional traits can control plant growth, productivity and interactions in forests^32^ and intraspecific trait variability can also drive species responses to climate change^33^. Thus, functional attributes of key plant species can be used to determine shifts between communities after fire along fine-scale environmental gradients such as the ones found along the coast-inland gradient.

Several studies have examined post fire succession on coastal dunes^34–36^. However, most of these works have focused primarily on comparing plant communities before and after the fire, without analysing the underlying mechanisms driving these shifts or accounting for the intrinsic coast-to-inland gradient characteristic of these habitats (but see^37^ resulting from the combination of biotic (e.g. plant cover and diversity, soil nitrogen availability, soil organic content) and abiotic (e.g., salt spray, high temperature, soil pH, sand transport) processes^38^. In this context, the present study aims to analyse the processes involved in the shifts in community structure and functional traits occurring after the fire, while considering the pre-existing plant community gradient affecting coastal dunes. To achieve this, this study examines an area impacted by a large wildfire in June 2017, exploring the relationships among key species and evaluating their functional trait responses and interactions along the coast-to-inland gradient. Specifically, this work aims to (i) determine whether post fire community dynamics vary along this gradient; (ii) understand the functional responses of dune shrubs to fire across the different zones; and (iii) study the role of species traits and community composition in shaping species interactions. Post fire communities are expected to follow a predictable successional trajectory, gradually resembling their pre-fire counterparts. However, the degree of similarity at any given time is likely to be influenced by the interplay between environmental and biotic factors shaping coastal dune ecosystems. In coastal areas, where the effects of environmental stressors are more intense, plant communities are likely to persist in earlier successional stages for an extended period. Conversely, inland communities, experiencing lower environmental constraints, are expected to progress through succession more rapidly, with biotic interactions exerting a greater influence on community dynamics.

## Methods

###  Study area

**Fig. 1:**
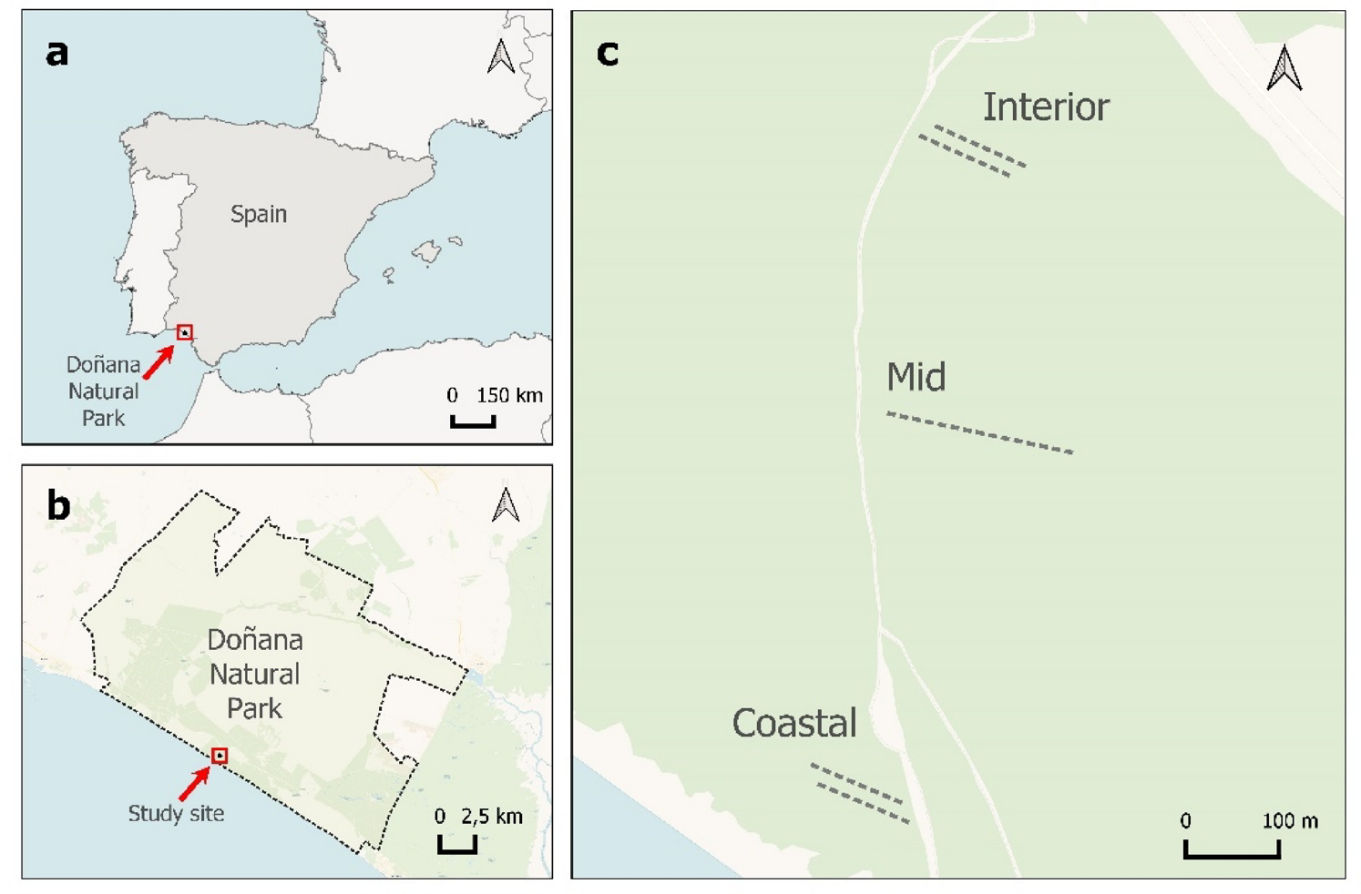
(**a**) Location of the study area in the southwest of Spain, along the Atlantic Ocean. (**b**) Boundaries of Doñana Natural Park, obtained from the *Plan de Ordenación de los Recursos Naturales del Espacio Natural Doñana* (Annex V)^48^. (**c**) Locations of the sampled transects. Maps were generated using QGIS (version 3.34.10)^49^, with Carto Voyager Labels (CC BY 4.0) as the basemap for panels (**b**) and (**c**).

This study was conducted in El Asperillo, a coastal dune system located in Doñana Natural Park (Huelva, SW Spain), covering about 30 km along the Atlantic coast with a width of 1 km in most areas (Fig. 1). This dune system includes the beach, young moving dunes, fossil dunes, and eolian sand sheets. Dunes can reach up to 60 m in height and are located above the beach, resting on a vertical sandstone cliff^39^ .

The region has a Mediterranean climate with oceanic influence and mild temperatures (Csa according to Köppen classification)^40^. The mean annual temperature is 16.8 °C, with peak temperatures in July and August (33.5 °C) and the lowest in January (6.9 °C). Annual rainfall averages 550 mm but varies significantly (170–1028 mm over the past 25 years)^41^, with 80% occurring from October to March^42^. The study years experienced significantly lower-than-average precipitation, with the years classified as dry (412 mm in 2020–2021) and very dry (282.5 mm in 2021–2022)^43^.

The study area was affected by a wildfire in June 2017. The burned area was extensive, covering 10,900 ha, 88.4% of which included protected areas such as the “Médano del Asperillo”, part of the Doñana Natural Park^44^. Historical records for the study site indicate fire events of approximately 1,000 ha in 1626 and 1805, as well as two more recent fires in 1981 (300 ha) and 1985 (900 ha), culminating in the fire of 2017^45^. While the exact size of fires prior to the 20th century remains uncertain, it is evident that fires are recurrent at a scale of decades to centuries, and also that the 2017 fire was the most devastating in the area in at least a century. Pre-fire vegetation was composed mostly by plantations of *Pinus pinea* L. and a shrub community dominated by *Corema album* (L.) D.Don, *Cistus halimifolius* L., *C. calycinum* L., *C. salviifolius* L., *Stauracanthus genistoides (Brot.) Samp.*, *Cytisus grandiflorus* (Brot.) DC., *Osyris lanceolata* Hochst. & Steud., a hemiparasitic species of pine trees and scrubs^41,46^, and isolated patches of *Juniperus phoenicea* L^47^.

After the fire disturbance, *O*. *lanceolata* and *C*. *album* were the first two species to appear because of their regrowth ability. After the rains of the 2018 spring, pyrophyte seeder species started to thrive, including *C. halimifolius* and *C. salviifolius*. Four years after the fire, distinct vegetation patches developed from the coast to the interior (field observation). Close to the coast, a community dominated by *C. album* and *C. halimifolius* with *S. genistoides*, *C. grandiflorus*, and *S. rosmarinus*, occurred; however, both species showed lower cover inland, where individuals of *Lavandula stoechas* L. were also recorded.

### Sampling methodology

Three sampling zones were established along the coast-inland gradient. The first sampling area, the Coastal zone, located by the shoreline (37°04′17.45′′ N, 6°41′18.26′′ W), had the highest stress level for dune plants because it is heavily influenced by coastal processes such as salt spray, high temperature, and burying. The next area, named Mid zone (37°04′31.95′′ N, 6°41′19.52′′ W), was located in an interdunal depression and showed evidence of higher levels of sediments, ash, and humidity than coastal and inland areas. Finally, the Interior zone was established (37°04′41.86′′ N, 6°41′17.40′′ W) in a zone with lower humidity and winds.

The sampling was conducted in February-March 2021 and 2022 in three 200-m transects located parallel to the sea. In both the interior and coastal zones, the sample transects were divided into two parallel 100-m sections as it was not possible to establish continuous 200-meter transects with a homogeneous community composition in this area; however, in the mid zone,, a continuous 200 m transect was used for sampling (Fig. 1). Plant species composition and cover were recorded along the transects using the point-intercept method^50^. Each transect was sampled using 20 consequent point‑intercept sub‑transects (each one 10 m long). Within each sub‑transect, a 5‑mm‑diameter rod was inserted vertically (90° to the soil surface) at 50 cm intervals. At each interval, every contact with vascular plant species, lichens, mosses, litter, or bare soil was recorded. Percent cover for each category was calculated by dividing the number of contacts by the total number of pin drops within that sub‑transect. This design yielded a total of 400 point-intercept observations per zone per year. Given the significance of soil nutrient availability in shrub communities growing on sandy soils^25^, as well as the varying ash depositions observed in the study area (personal observation), soil samples were also collected during the 2021 surveys to characterize the soil composition along the coast-inland gradient. Soil samples were collected from two points along each transect: one at the beginning and one at the end. These samples were taken from the top 20 cm of soil and then combined to create a single composite sample for each transect, in accordance with the standard practices employed by other research teams working in Doñana^51^. Soil samples were analysed for particle size (% of sand, silt, and clay), humidity, organic carbon, organic matter, total Kjeldahl nitrogen, Olsen phosphorus, potassium, calcium and magnesium at IRNAS-CSIC (Seville, Spain).

### Functional diversity

To characterise the community responses to the fire of the studied communities and their variation along the coast to inland gradient, eight species, *C. album*,* C. halimifolius*,* C. calycinum*,* C. salviifolius*,* S. genistoides*,* C. grandiflorus*,* O. lanceolata* and *S. rosmarinus*, were selected according to their abundance and functional relevance in Iberian Atlantic plant dune communities^46,52,53^. In each zone, five individuals per species were selected for measuring different morphological traits (Table S1) such as plant height (h) and maximum and minimum orthogonal diameters (DM and Dm) of plant canopy. Additionally, for each plant, a sample of fresh leaves was carried in a refrigerated box to the laboratory, where leaf area (LA), turgor and dry weights were measured to calculate different ecophysiological leaf traits such as LMA (Leaf Mass Area), LDMC (Leaf Dry Matter Content) and SLA (Specific Leaf Area). LMA is the inverse of SLA and quantifies the ratio between leaf area (LA) and leaf dry weight. Both are good indicators of plant functioning parameters such as photosynthetic rates, resistance to herbivory, etc^54^. LDMC is the ratio between the turgor weight and the dry weight, and it is associated with the trade-off between efficient nutrient conservation (high LDMC) and fast biomass production (low LDMC)^55^. To calculate LMA and SLA, the LA of 10 leaves per species were measured, except for *C*. *album*, where the leaves present in 3–4 cm branch segments were used, and *S. genistoides* and *C. grandiflorus*, in which terminal branches of 3–4 cm long were measured as they represent the photosynthetic tissue (see^56^ for details). LA was assessed using Easy Leaf Area Free (v. 1.02), an app for mobile devices, with a standardized setup in the lab. Then, the samples were preserved at 4°C, in darkness and with full humidity (inside zip bags with some drops of water) for 24 h to create full turgor in the samples. After this time, and without extending it further to prevent fungal growth, the samples were dried to eliminate the exterior water in the leaf with paper and then weighted. After that, the samples were placed in paper envelopes, dried at 75°C for 24 h, and cooled for about 8–12 h in the oven. This process helped to prevent the samples from reabsorbing moisture from the air as they cooled. Then, samples were weighted again to obtain the leaf dry weight^57^.

For each plant, the area of the canopy projection and volume of the plants was estimated as seen in Eqs. 1 and 2:

1 $$Area=\:\frac{DM}{2}*\:\frac{Dm}{2}*\:\pi\:$$

2 $$Volume=\:\frac{4\pi\:}{3}*h*DM*Dm$$

DM = mayor diameter, Dm = minor diameter, h = height.

Finally, in order to assess the impact of the main different strategies developed by plants to persist after the fire, the eight species were classified into the resprouter (individuals that resprout from surviving parental tissues) and seeder (individuals that die but release seeds able to germinate) functional groups^57,58^.

Trait variations between communities were described by the community-weighted mean of each trait measured (CWM), defined as the mean of trait values present in the community weighted by the relative abundance of the species bearing each value^59^, CWM values were first calculated using the function cwm of R Package “weimea”^60^. Then, a Principal Component Analysis (PCA) was performed with the CWM trait values in each transect to define the main functional trends in the community and to avoid collinearity^61^, using the function rda of R Package “vegan”. To calculate the contribution of each trait to the PCA axes, the function “fviz_contrib” of R Package “factoextra”^62^ was used. After that, one-way ANOVAs were performed to confirm the main trait trends among communities identified by the PCA using the ‘‘rstatix’’^63^ R Package. Analyses were performed to check outliers and confirm normality and homogeneity. Finally, Tukey post-hoc tests were used to perform multiple pairwise comparisons between the three communities.

### Community assembly

Plant cover data were grouped at 10-, 20-, and 40-m clusters in order to generate a co-occurrence matrix at various scales and analyse the community assembly dynamics^64^. Variations in assemblage composition were described with a Non-Metric Multidimensional Scaling ordination (NMDS) of the 120 (60 per year) 10-m sampling transects based on their shrub cover with the function metaMDS of R Package “vegan”^65^. Data were transformed using Wisconsin double standardization, in which the abundance of each species in each plot is first divided by the maximum observed value for that species, emphasizing rare species, and then divided by the total abundances for all species in the plot, indicated when sampling occurs across multiple years^65,66^.The Bray-Curtis method was used to measure the distance/similarity between plots. Both NMDS and Bray-Curtis distance are preferably used in community analysis because they deal better with null values^67^.

Indicator species serve as ecological marks of a certain community since they ultimately integrate the qualitative characteristics of the ecosystem^68^. Thus, the indicator species were identified in the three communities by calculating all species’ indicator values (IndVal^69^. This index quantifies the fidelity and specificity of each species to a given type of community. The “indicspecies” R Package was used to calculate the IndVal values^70^.

Finally, to assess the main drivers affecting community composition and analyse trait dynamics, the relationship between the NMDS ordination and two main factors, namely distance to the coast and sampling year, and the PCA axes obtained from the CWM trait values analysis, as proxies of main functional trends, were examined through vector fitting. Then, those variables presenting significant correlations were overlaid in the NMDS ordination^71^.

### Spatial structure of the community

A co-occurrence network was constructed to analyze the spatial structure of the woody plant community within each transect. Community detection was performed using the “igraph” package in R^72^, which provides various algorithms for detecting community structures in networks^73^. To assess the community structure of our network, the Louvain algorithm, a multi-level modularity optimization method designed to detect communities^74^, was applied. This algorithm has been shown to perform efficiently on networks with fewer than 1,000 nodes^75,76^. The modularity of the resulting partitions was then computed to assess the strength of the community organization. Modularity quantifies the density of links within communities compared to links between them^75^. This process was repeated for each combination of area and year, allowing us to compare community structures across different spatial and temporal scales.

Finally, to further investigate the spatial structure of the studied community, Moran’s I was used to assess spatial autocorrelation using the “spdep”^77^ and “geosphere”^78^ packages in R. Three approaches were applied: first, species-level patterns were analyzed per year and area along linear transects using rook’s case neighbors and row-standardized weights; second, total woody plant abundance was assessed using the same linear structure; and third, using the transect coordinates with sampling points each 10 m to compute Moran’s I using 2-nearest-neighbor spatial weights.

## Results

### Study area characterization

According to the soil parameter analyses, soil texture was sandy in all samples (97.8–100% sand, 0–2.3% silt and zero clay), but the coastal area was the most arid and impoverished zone, followed by the interior, while the mid zone had the greatest concentrations and variety of nutrients (Table 1). While increasing the number of soil samples and/or conducting repeated sampling in 2022 would likely enhance the robustness of these results, the existing values clearly indicate that the mid zone exhibits the highest levels of both nutrients and humidity. Species number did not show large differences between transects, although the mid zone presented the highest number of species. Compared to the 10 species on average for each 200 m transect in the mid zone, transects in the coastal zone hosted an average of seven species in 2021 and eight species in the following year, and the interior ones had nine species on average. These results suggest a small variation in species richness among the zones, with the mid zone consistently showing a slightly higher number of species compared to the other zones.

**Table 1:** Soil composition and parameters in the different zones.

Zone	Humidity (over wet weight, %)	Organic carbon (%)	Organic matter (%)	Kjeldahl nitrogen (%)	Olsen phosphorus (mg/kg)	K (mg/kg)	Ca (mg/kg)	Mg (mg/kg)
Coastal 1	0.4	0.200	0.34	0.008	1.1	25.4	103	30.1
Coastal 2	0.2	0.213	0.37	0.005	1.2	12.3	60	20.4
Mid 1	**1**.**7**	**3**.**070**	**5**.**29**	**0**.**185**	**7**.**9**	**52**.**2**	**1436**	**234**
Mid 2	**0**.**5**	**0**.**941**	**1**.**62**	**0**.**046**	**1**.**6**	33.6	**377**	**92**.**4**
Interior 1	0.4	0.091	0.16	0.012	< 1	23.8	108	27.4
Interior 2	0.2	0.566	0.98	0.034	1.4	**34**.**0**	229	59.2

The two highest values were marked in bold.

### Functional diversity

**Fig. 2:**
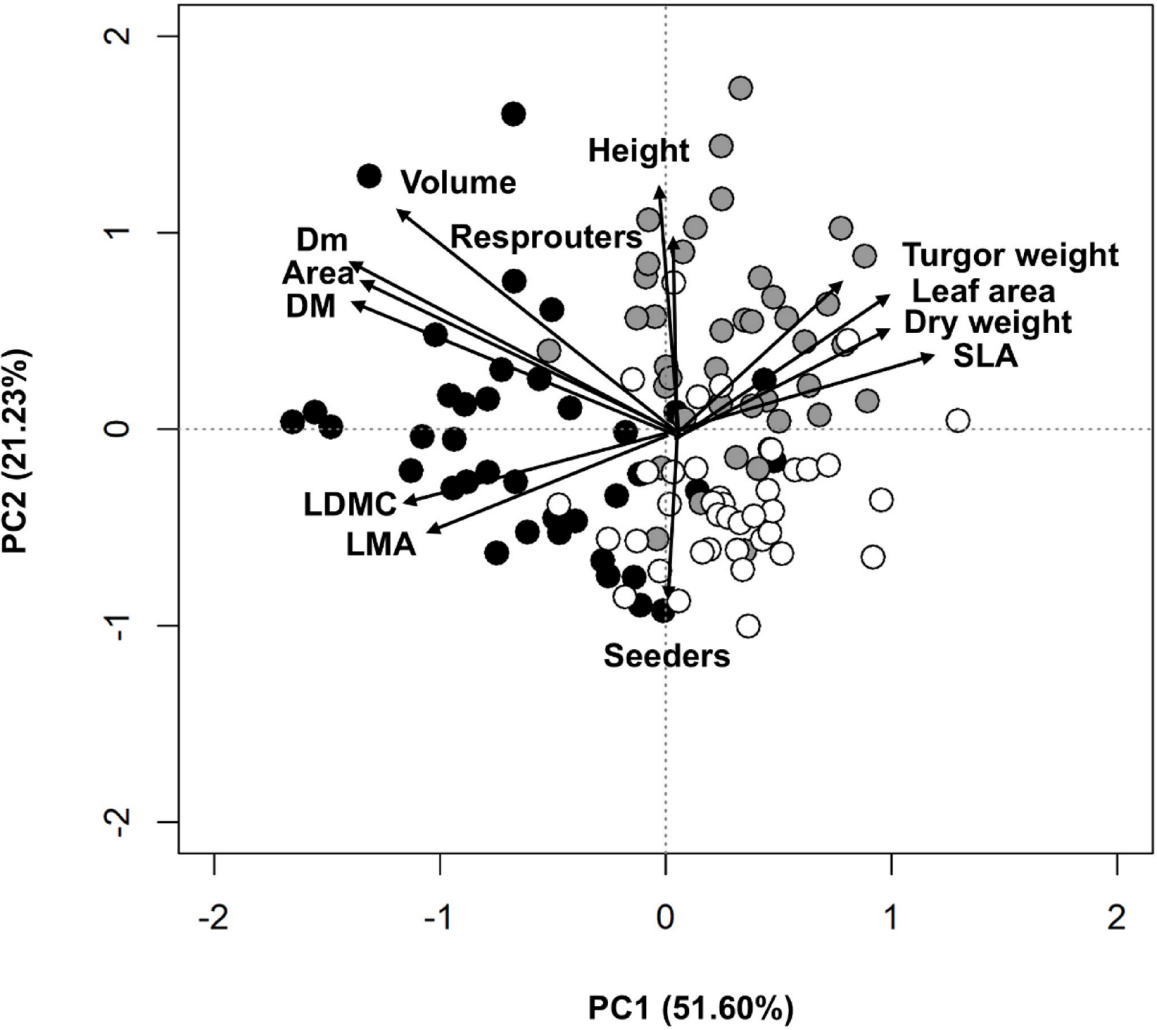
Axes 1 and 2 of the PCA ordination of transects are based on the community weighted mean of each trait measured (CWM). Black symbols represent transects located near the coast, grey symbols in the mid zone and white in the interior zone. Arrows reflect the main variables identified by the correlation analyses.

Regarding the functional trait variation at the community level, in the PCA performed with the CWM trait values in each transect, the first two axes were selected for ulterior statistical analyses after considering their eigenvalues, explained variance and (existing or lack of) ecological meaning. Axis 1 (herein PC1) represented 51.60% of the variance and reflects a gradient of scrub sclerophylly (> 60% contribution; Fig. 2; Table 2). Axis 2 (PC2) represented 21.23% of the variance and accounts for a plant size gradient (40% contribution) and, secondarily, for the dominance of seeders or resprouters (10% contribution). The PCA identified structural (plant area, height, and volume), ecophysiological (leaf area, LMA, and SLA), and ecological (percentage of seeders and resprouters) traits as defining descriptors of the main functional dynamics within the community. One-way ANOVAs and Tukey tests revealed that plants in coastal and interior zones exhibited greater sclerophylly, with higher LMA values, whereas those in the mid zone displayed larger leaf area and SLA. Additionally, plants in the mid zone attained the greatest canopy height, while coastal plants had the largest area and volume (Table 2). Finally, the resprouter cover was significantly higher in the mid zone.

**Table 2:** Mean and standard deviation of (i) the values of the plant traits summarizing the main functional trends of the shrub communities and (ii) the axes of the multivariate ordinations performed (PCA and NMDS) by zone: coastal, mid, and interior.

Traits	Coastal	Mid	Interior
Plant area (m^2^)	**1.11 ± 0.33 a**	0.64 ± 0.21 b	0.49 ± 0.16 c
Plant height (cm)	72.40 ± 12.60 a	**91.50 ± 12.80 b**	69.40 ± 8.56 a
Plant volume (m^3^)	**5.46 ± 1.90 a**	4.17 ± 1.98 b	2.39 ± 1.22 c
Leaf Area (cm^2^)	6.15 ± 2.62 a	**12.1 ± 2.47 b**	11.0 ± 2.88 b
LMA (g/m^2^)	**371 ± 58.6 a**	303 ± 55.2 b	350 ± 55.1 b
SLA (m^2^/kg)	3.2 ± 6.48 a	**4.19 0.62 b**	3.73 ± 0.6 c
Seeder cover (%)	80.2 ± 0.22.8 a	65.5 ± 24.8 b	**82.4 ± 14.8 a**
Resprouter cover (%)	19.8 ± 22.8a	**34.5 ± 24.8b**	17.6 ± 14.8 a
PC1	-0.09 ± 0.08 a	0.05 ± 0.05 b	0.05 ± 0.06 b
PC2	-0.01 ± 0.09 a	0.07 ± 0.08 b	-0.05 ± 0.06 c
NMDS1	-0.60 ± 0.24 a	0.39 ± 0.34 b	0.21 ± 0.31 c
NMDS2	-0.05 ± 0.46 a	-0.02 ± 0.32 a	0.07 ± 0.38 a

Letters denote significant differences identified by Tukey’s post hoc tests for pairwise differences across the three zones.

The highest value of each plant trait is marked in bold.

### Community assembly

**Fig. 3:**
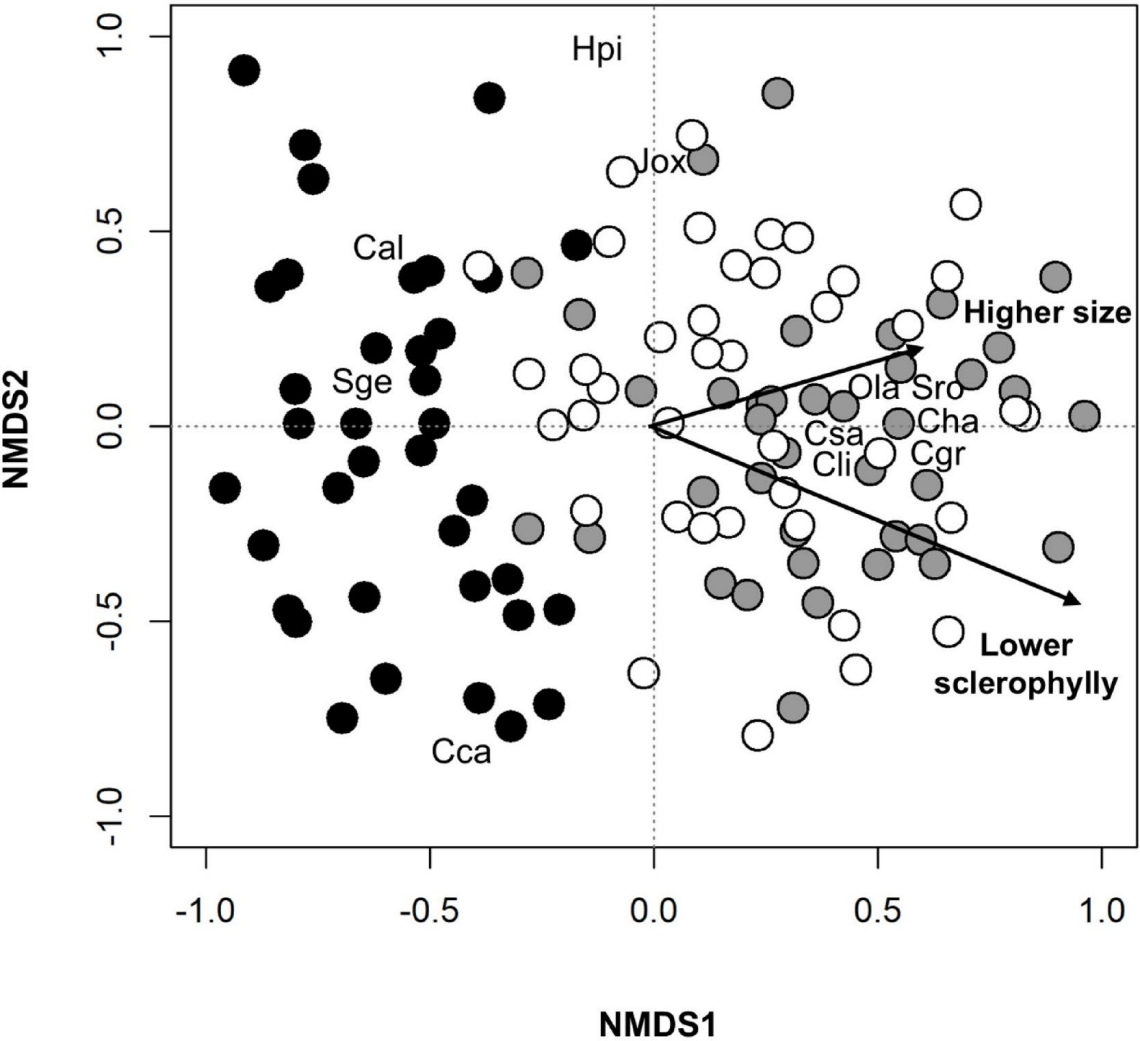
Axes 1 and 2 of the 2-dimensional non-metric multidimensional scaling ordinations of transects based on shrub cover (NMDS1 and NMDS2). Black symbols represent transects located near the coast, grey symbols in the mid location and white in the interior area. Arrows reflect the main gradients identified by the correlation analyses. The species identified in the analysis are: Cal: *Corema album;* Sge: *Stauracanthus genistoides;* Cca: *Cistus* calycinum; Hpi: *Helychrisum* picardii; Jox: *Juniperus oxycedrus;* Csa: *Cistus salviifolius;* Ola: *Osyris lanceolata;* Cha: *Cistus halimifolius;* Sro: *Salvia rosmarinus;* and Cgr: *Cytisus grandiflorum.*

The main variations in species composition of sampled transects were described by the two first axes of an NMDS, with a final stress value of 0.23. The first NMDS axis identifies a gradient from the coastal zone, which is distributed along the negative values of the axis, to the mid and interior communities, which are clustered along the positive ones (Fig. 3). The coastal zone is dominated by *C. album* and *S. genistoides*, while the other communities of the positive axis 1 are dominated by Cistaceae species such as *C. halimifolius* and *C. salviifolius*, together with *O. lanceolata*, *S. rosmarinus* and *C. grandiflorum*. The relationship between these five species and the middle and interior communities were confirmed by the indicator value (IndVal) analysis. Although no species with a significant IndVal for a single community were found, these five species presented significant indicator values for the groups middle and interior vs. coastal: *C. halimifolius* (0.951, *p* < 0.001), *C. salviifolius* (0.882, *p* < 0.001), *C. grandiflorum* (0.880, *p* < 0.001), *S. rosmarinus* (0. 563, *p* < 0.001), and *O. lanceolata* (0.461, *p* < 0.002).

Vector fitting confirmed the correlation between the communities described by the NMDS ordination and (i) the distance of the communities to the coast (r^2^ = 0.45, *p* < 0.001), (ii) the shrub sclerophylly gradient (r^2^ = 0.63, *p* < 0.001), described by the PC1 axis and (iii) the size of the shrubs (r^2^ = 0.18, *p* < 0.001), described by the PC2 axis (Fig. 3, Table S2). Furthermore, the sample year and the abundance of resprouters and seeders showed no clear effect on the dynamics of shrub community composition.

### Spatial structure of the community

**Fig. 4:**
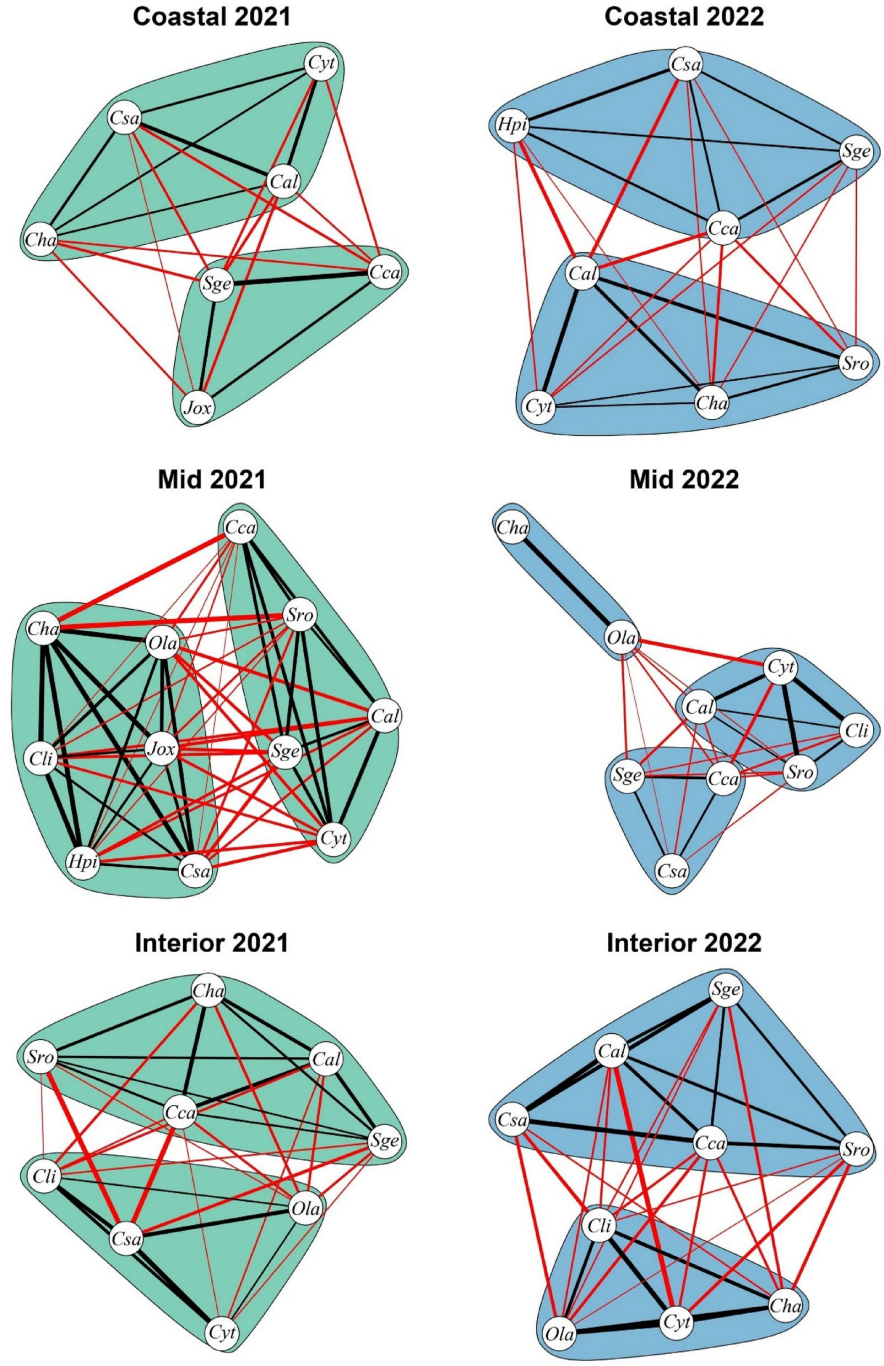
Variation in the spatial structure of the communities within the three sampled areas. Each circle represents one species; the thickness of the line represents the weight of the interaction (in this case, degree of co-occurrence based on the adjacency ratio matrix); and the colour if the plant is interacting with a species of the same group (black) or not (red). Species codes as in Fig. 3.

The modularity analysis showed how the organization of the species networks changed between years and ecological zones (Table 3; Fig. 4). In the coastal zone, two groups were detected in both years, with a slight increase in species richness and a modest rise in modularity, which suggests a mild increase in the spatial clustering of species over time. In the mid zone, there was a decrease in species richness, but both the number of groups and the modularity value increased, indicating an enhancement in community structure, with more distinct clusters of species forming despite the lower richness. In the interior zone, species richness remained stable, and two groups were detected in both years. Modularity values were also similar, suggesting that the spatial structure of the community remained consistent over time, with no clear gain or loss in clustering.

**Table 3:** Species richness and modularity values of the three sample zones in Asperillo (Coastal, mid zone, and Interior) for the years 2021 and 2022. The table displays the number of species and groups detected in each zone, as well as the corresponding modularity values obtained using the Louvain method.

Zone	2021			2022		
	Composition		Modularity	Composition		Modularity
Coastal	Species	7	0.016	Species	8	0.042
	Groups	2		Groups	2	
Mid	Species	11	0.017	Species	9	0.130
	Groups	2		Groups	3	
Interior	Species	9	0.060	Species	9	0.057
	Groups	2		Groups	2	

Regarding the spatial structure of woody plant distributions, Moran’s I analyses revealed that across the 53 species–zone possible combinations (limited by the presence of individuals in the transects), seven showed statistically significant spatial autocorrelation, six indicating aggregation and one segregation, while the remaining combinations exhibited random spatial patterns (Table S3). When analyzing total woody plant abundance per transect, no significant spatial structure was detected in any zone (Table S4), suggesting randomness in individual distributions. Likewise, incorporating geographic coordinates into the analysis did not reveal any significant spatial autocorrelation (Table S5; Figure S1), reinforcing the overall absence of spatial structuring across the system.

## Discussion

Overall, the findings of this work indicate that natural secondary succession in the study area has led to the development of a set of gradient-dependent shrub communities resembling those that existed prior to the fire, pointing to a consistent pattern of recovery^39,47^. This indicates that community responses after the fire are predictable from a set of underlying mechanisms that link regional species pool, regeneration traits and physical heterogeneity^79^. Moreover, although community assembly analyses indicate that coastal community exhibited a significant taxonomic divergence from mid and interior communities, reflecting their overall differential responses to environmental drivers, functional trends exhibit more complex and contrasting patterns. Traits associated with sclerophylly, such as LMA, SLA, and LA, differentiate the coastal zone from the interior and mid zones, whereas size-related traits, namely area and volume of plants, distinguish all three zones. Finally, seedling recruitment constitutes the primary post fire regeneration strategy in these emerging communities, reflecting their higher colonization ability^80^, though its expression is significantly lower in the mid zone. Interestingly, four years after the fire, two keystone species of the current coastal dune system of Doñana Natural Park, *Pinus pinea* (planted in the 1950s) and the native *Juniperus phoenicea*, have neither germinated nor sprouted.

Coastal habitats are known for their harsh environmental conditions, making functional traits essential for understanding how these factors directly impact plant fitness and, in turn, shape plant community composition^33^. At our study site, plants in the coastal areas exhibited higher LMA values, indicating more efficient water use^81^. Higher LMA scores in the coastal zone plots are associated with the dominance of *C. album* and *S. genistoides*. As *C. album* leaves are folded to the underside, the dry matter is higher per surface area, which is reflected in LMA values. Besides, *C. album* has other adaptations such as a thick cuticula, stomata buried in the folded area protected by hairs, perfectly efficient stoma control and the capacity to save water, making this species well-adapted to this kind of dry environments^82,83^. Additionally, *S. genistoides* is a legume which has no leaves, and its young spiny branches constitute the photosynthetic organs^84^. The plots of the coastal area also exhibit higher scores of plant area and volume, morphological characteristics associated to the instability of coastal dunes and the effect of winds, which favours the horizontal spread and the adoption of a cushion-shape of the plants in response to sand deposition or erosion^85^. In contrast, plants from the mid and interior zones exhibited higher SLA and LA values, reflecting a distinct functional response closely tied to the varying influence of abiotic factors that filter the coastal dune species pool^86^. While the strongest trait selection occurs in the coastal zone, this filtering effect is less pronounced in the mid zone, where plants tend to grow taller. This enhanced plant development is driven by greater nutrient availability, increased soil moisture, and a reduced impact of environmental stressors, fostering an environment where biotic interactions play a more significant role in shaping community structure^38^. Moreover, the higher SLA and LA values are primarily linked to the dominance of Cistaceae species^87^, while increased plant height in the mid transects indicates a secondary gradient favoring greater growth. This secondary gradient is exemplified by *Cistus halimifolius*, the species with the highest indicator value for these communities. Previous studies have shown that *C. halimifolius* is highly plastic, readily germinating after fire and adapting its morphology and physiology to prevailing environmental conditions^88,89^. Therefore, *C. halimifolius*, and the four other species with significant indicator values for both communities: *C. salviifolius*,* C. grandiflorum*,* S. rosmarinus* and *O. lanceolata*) respond to the higher nutrient and humidity levels in the mid zone.

Furthermore, after five years, there is a clear selection for seeders such as *C. halimifolius*,* C. salviifolius*,* S. genistoides*,* C. calycinum*,* C. grandiflorum* and *S. rosmarinus*, as the predominant fire-response strategy. In Mediterranean ecosystems, relict Tertiary species with ancestral traits, such as obligate resprouters, can often persist through the facilitative effects of species with Quaternary traits, such as obligate seeders^90^. Thus, a diversity of regeneration niches is maintained, contributing to the remarkable biodiversity characteristic of these ecosystems^91^. The overall dominance of seeder species across the study system reflects both the harsh conditions of these coastal environments and the superior colonization capacity of seeders^80^. Moreover, the significant increase in resprouter cover within the mid zone, namely *C. album* and *O. lanceolata*, appears to be driven by the more favorable edaphic conditions of this interdunal depression, particularly higher soil moisture, as resprouting individuals are more susceptible to dehydration^92^.

Additionally, our results evidence that community composition does not present clear differences between 2021 and 2022 sampling dates. However, the modularity analysis reveals distinct differences in their community structure. Based on the modularity scores, the interior site presents lower values of species clustering, which hints a diffuse community structure without strong spatial associations between species^93^. On the other hand, biotic interactions seemingly have a more prominent role on the mid site and may be responsible for the clusters of species observed in the modularity analysis. Moreover, the coastal site also shows clusters of species, which could be attributed to stronger environmental filtering effects and the presence of biotic interactions. Here it is important to stress that, although the Louvain method has already been used to analyze co-occurrence networks^94–96^, its potential in studying community structure remains largely underexplored. This work demonstrates how this approach can provide valuable insights into community organization, offering a quantitative framework to detect species associations and assess the processes shaping ecological communities. By applying network-based modularity analysis, it’s possible to benefit from a powerful tool to uncover hidden patterns of species organization and interaction, improving our understanding of the factors driving community structure.

Finally, the results of our interannual comparison — namely, the persistence of similar species compositions alongside increasing modularity in the coastal and mid zones, the growing group complexity in the mid zone, and the stable structure in the interior zone — suggest that these communities are progressing beyond the initial stages of environmental filtering. Hence, most of the species capable of resprouting and growing, regardless of the abiotic stress, have successfully colonized the area. Currently, community structure is starting to be shaped by biotic interactions, which operate at a smaller spatial scale to determine the spatial arrangement of the community. This suggests that the community assembly process is currently at an intermediate stage of succession. In other words, the community is now transitioning towards a phase where biotic interactions become more important in shaping community structure. As such, changes in structure and composition are expected to occur over time as the community continues to evolve and adjust to the prevailing environmental conditions and biotic interactions^4,46,8^. Accordingly, further studies analysing biotic interactions between species over longer time scales could significantly enhance our understanding of successional dynamics in post-fire plant community assembly within Mediterranean coastal dunes.

## Conclusions

Natural secondary succession in the study area has resulted in the formation of gradient-dependent shrub communities that resemble those present before the fire. This highlights the predictability of post fire community responses through mechanisms connecting the regional species pool, regeneration traits, and physical heterogeneity. It also underscores the significance of environmental filters and species interactions in shaping community assembly.

Five years after the wildfire, the study area progresses through advanced ecological succession, with early interactions emerging among plant species. Although the communities have not yet returned to their original state, they continue to develop with increasing complexity in both functionality and spatial structure. A comparison of data from 2021 to 2022 shows annual changes, especially in the interior zones, suggesting the value of ongoing monitoring to capture further shifts. These findings could offer key insights for managing similar ecosystems. Moreover, although the 2017 fire created a unique opportunity to observe natural succession without planted pines, another fire would challenge resprouting species, as they may not yet have stored sufficient reserves in their underground organs, potentially leading to their decline. Increased fire frequency and severity could reduce the abundance of resprouters, like *C. album*, and lead to a homogenization of plant communities.

## Electronic supplementary material

Below is the link to the electronic supplementary material.

Supplementary Material 1.

## Data Availability

The datasets used and/or analysed during the current study available from the corresponding author on reasonable request.
