# Supplementary material for: Dynamics of post fire plant community assembly in Doñana coastal dunes
Source: Sci Rep. 2025 Jun 6;15:19935. doi: 10.1038/s41598-025-04400-x (PMC12144271; doi:10.1038/s41598-025-04400-x)

**SUPPORTING INFORMATION**

1. **Dynamics of post fire plant community assembly in Doñana coastal dunes**
2. Sergio Chozas, André F. Mira, Manuel Serrano, Nagore G. Medina, Joaquín Hortal and María Cruz Díaz-Barradas

**Table S1.** Values of the functional traits measured in five individuals of eight species in each zone (coastal, mid and inland)

**Codes**: plant **height**, maximum and minimum orthogonal diameters (**DM** and **Dm**), **Area** and the **Volume** of each individual, **LMA** (Leaf Mass Area), **LDMC** (Leaf Dry Matter Content) and **SLA** (Specific Leaf Area), leaf area (**LA**) and leaf dry weight (**LDW**)

*Corema album* (**Cal**), *Cistus halimifolius* (**Cha**), *C. calycinum* (**Cca**), *Cistus salviifolius* (**Csa**), *Stauracanthus genistoides* (**Sge**), *Cytisus grandiflorus* (**Cgr**), *Osyris lanceolata* (**Ola**) and *Salvia rosmarinus* (**Sro**).

**FG**: Functional groups (seeders and resprouters)

| **ID** | **species** | **zone** | **DM**  **(cm)** | **Dm**  **(cm)** | **Height**  **(cm)** | **Area**  **(cm^2^)** | **Vol (cm^3^)** | **LA (cm^2^)** | **Turgor**  **(g)** | **LDW**  **(g)** | **LDMC**  **(mg/g)** | **LMA**  **(g/m2)** | **SLA**  (**m^2^/kg**) | **FG** |
| --- | --- | --- | --- | --- | --- | --- | --- | --- | --- | --- | --- | --- | --- | --- |
| Cal1 | Cal | coastal | 225 | 160 | 85 | 28274.33 | 12817698 | 2.34 | 0.25 | 0.13 | 517.17 | 547.01 | 1.83 | Resprouter |
| Cal2 | Cal | coastal | 130 | 120 | 65 | 12252.21 | 4247433 | 2.51 | 0.3 | 0.16 | 521.93 | 621.12 | 1.61 | Resprouter |
| Cal3 | Cal | coastal | 195 | 190 | 115 | 29099 | 17847388 | 2.74 | 0.24 | 0.12 | 482.52 | 428.1 | 2.34 | Resprouter |
| Cal4 | Cal | coastal | 140 | 135 | 95 | 14844.03 | 7520973 | 3.12 | 0.23 | 0.12 | 519.51 | 383.97 | 2.6 | Resprouter |
| Cal5 | Cal | coastal | 115 | 55 | 65 | 4967.64 | 1722116 | 2.9 | 0.24 | 0.11 | 468.03 | 393.79 | 2.54 | Resprouter |
| Cha1 | Cha | coastal | 190 | 150 | 170 | 22383.85 | 20294689 | 17.98 | 0.92 | 0.36 | 392.22 | 200.89 | 4.98 | Seeder |
| Cha2 | Cha | coastal | 145 | 125 | 195 | 14235.34 | 14804755 | 26.4 | 1.65 | 0.63 | 381.3 | 237.88 | 4.2 | Seeder |
| Cha3 | Cha | coastal | 115 | 110 | 105 | 9935.29 | 5563761 | 14.15 | 0.59 | 0.22 | 365.1 | 152.72 | 6.55 | Seeder |
| Cha4 | Cha | coastal | 155 | 110 | 125 | 13391.04 | 8927359 | 18.7 | 0.82 | 0.36 | 434.44 | 189.95 | 5.26 | Seeder |
| Cha5 | Cha | coastal | 190 | 120 | 110 | 17907.08 | 10505486 | 20.11 | 1.03 | 0.38 | 367.14 | 188.46 | 5.31 | Seeder |
| Csa1 | Csa | coastal | 145 | 140 | 65 | 15943.58 | 5527109 | 12.2 | 0.98 | 0.29 | 297.07 | 237.46 | 4.21 | Seeder |
| Csa2 | Csa | coastal | 175 | 130 | 120 | 17867.81 | 11435397 | 35.17 | 2.54 | 0.76 | 298.29 | 215.75 | 4.63 | Seeder |
| Csa3 | Csa | coastal | 75 | 70 | 70 | 4123.34 | 1539380 | 34.32 | 2.41 | 0.77 | 319.95 | 224.8 | 4.45 | Seeder |
| Csa4 | Csa | coastal | 80 | 70 | 90 | 4398.23 | 2111150 | 25.64 | 2.26 | 0.71 | 312.56 | 275.86 | 3.63 | Seeder |
| Csa5 | Csa | coastal | 70 | 65 | 35 | 3573.56 | 667064.8 | 21.3 | 1.56 | 0.43 | 274.63 | 200.52 | 4.99 | Seeder |
| Sge1 | Sge | coastal | 140 | 105 | 70 | 11545.35 | 4310265 | 5.75 | 0.38 | 0.18 | 468.73 | 306.26 | 3.27 | Seeder |
| Sge2 | Sge | coastal | 110 | 80 | 60 | 6911.5 | 2211681 | 6.13 | 0.45 | 0.22 | 499.11 | 365.25 | 2.74 | Seeder |
| Sge3 | Sge | coastal | 55 | 50 | 45 | 2159.84 | 518362.8 | 8.16 | 0.87 | 0.42 | 482.85 | 514.09 | 1.95 | Seeder |
| Sge4 | Sge | coastal | 95 | 75 | 80 | 5595.96 | 2387610 | 7.49 | 0.71 | 0.36 | 500.63 | 477.44 | 2.09 | Seeder |
| Sge5 | Sge | coastal | 145 | 145 | 95 | 16513 | 8366585 | 7.88 | 0.71 | 0.36 | 508.82 | 457.36 | 2.19 | Seeder |
| Cca1 | Cca | coastal | 35 | 30 | 30 | 824.67 | 131946.9 | 2.84 | 0.18 | 0.06 | 328.13 | 207.39 | 4.82 | Seeder |
| Cca2 | Cca | coastal | 40 | 35 | 35 | 1099.56 | 205250.7 | 3.97 | 0.26 | 0.09 | 335.52 | 218.64 | 4.57 | Seeder |
| Cca3 | Cca | coastal | 55 | 35 | 35 | 1511.89 | 282219.7 | 4.77 | 0.28 | 0.08 | 282.14 | 162.89 | 6.14 | Seeder |
| Cca4 | Cca | coastal | 55 | 40 | 35 | 1727.88 | 322536.9 | 3.44 | 0.19 | 0.06 | 313.27 | 170.93 | 5.85 | Seeder |
| Cca5 | Cca | coastal | 35 | 35 | 30 | 962.11 | 153938 | 3.76 | 0.24 | 0.08 | 318.28 | 202.39 | 4.94 | Seeder |
| Ola1 | Ola | coastal | 220 | 200 | 230 | 34557.52 | 42390557 | 23.76 | 1.27 | 0.45 | 357.6 | 190.82 | 5.24 | Resprouter |
| Ola2 | Ola | coastal | 190 | 180 | 140 | 26860.62 | 20055928 | 12.3 | 0.79 | 0.31 | 396.42 | 255.61 | 3.91 | Resprouter |
| Ola3 | Ola | coastal | 175 | 165 | 115 | 22678.37 | 13909401 | 27.21 | 2.03 | 0.68 | 337.77 | 251.56 | 3.98 | Resprouter |
| Ola4 | Ola | coastal | 215 | 180 | 150 | 30394.91 | 24315927 | 19.31 | 0.8 | 0.25 | 307.19 | 127.81 | 7.82 | Resprouter |
| Ola5 | Ola | coastal | 225 | 205 | 160 | 36226.49 | 30913272 | 30.11 | 1.47 | 0.53 | 361.47 | 176.19 | 5.68 | Resprouter |
| Cgr1 | Cgr | coastal | 110 | 110 | 90 | 9503.32 | 4561593 | 6.1 | 0.75 | 0.33 | 441.92 | 544.43 | 1.84 | Seeder |
| Cgr2 | Cgr | coastal | 150 | 135 | 120 | 15904.31 | 10178760 | 4.7 | 0.64 | 0.25 | 398.65 | 539.36 | 1.85 | Seeder |
| Cgr3 | Cgr | coastal | 160 | 160 | 120 | 20106.19 | 12867964 | 6.05 | 0.47 | 0.14 | 306.82 | 237.19 | 4.22 | Seeder |
| Cgr4 | Cgr | coastal | 120 | 110 | 95 | 10367.26 | 5252743 | 8.7 | 1.23 | 0.54 | 439.72 | 619.15 | 1.62 | Seeder |
| Cgr5 | Cgr | coastal | 100 | 110 | 90 | 8639.38 | 4146902 | 7.03 | 0.59 | 0.2 | 335.42 | 282.36 | 3.54 | Seeder |
| Sro1 | Sro | coastal | 55 | 45 | 65 | 1943.86 | 673871.6 | 4.9 | 0.34 | 0.11 | 322 | 226.12 | 4.42 | Seeder |
| Sro2 | Sro | coastal | 60 | 35 | 70 | 1649.34 | 615752.2 | 3.57 | 0.28 | 0.09 | 325.59 | 255.18 | 3.92 | Seeder |
| Sro3 | Sro | coastal | 40 | 40 | 65 | 1256.64 | 435634.2 | 4.63 | 0.28 | 0.1 | 345.64 | 206.26 | 4.85 | Seeder |
| Sro4 | Sro | coastal | 80 | 50 | 100 | 3141.59 | 1675516 | 3.24 | 0.18 | 0.07 | 358.46 | 204.01 | 4.9 | Seeder |
| Sro5 | Sro | coastal | 40 | 30 | 45 | 942.48 | 226194.7 | 2.56 | 0.17 | 0.05 | 316.58 | 214.06 | 4.67 | Seeder |
| Cal6 | Cal | mid | 70 | 35 | 55 | 1924.23 | 564439.5 | 2.53 | 0.17 | 0.07 | 435.43 | 290.51 | 3.44 | Resprouter |
| Cal7 | Cal | mid | 80 | 65 | 70 | 4084.07 | 1524720 | 1.86 | 0.14 | 0.07 | 486.58 | 370.43 | 2.7 | Resprouter |
| Cal8 | Cal | mid | 70 | 65 | 45 | 3573.56 | 857654.8 | 2.35 | 0.19 | 0.1 | 520.17 | 411.49 | 2.43 | Resprouter |
| Cal9 | Cal | mid | 65 | 55 | 50 | 2807.8 | 748746.3 | 2.8 | 0.31 | 0.14 | 434.13 | 486.07 | 2.06 | Resprouter |
| Cal10 | Cal | mid | 75 | 70 | 45 | 4123.34 | 989601.7 | 1.05 | 0.09 | 0.05 | 511.95 | 428.57 | 2.33 | Resprouter |
| Cha6 | Cha | mid | 75 | 70 | 110 | 4123.34 | 2419026 | 11.95 | 0.62 | 0.23 | 374.52 | 195.82 | 5.11 | Seeder |
| Cha7 | Cha | mid | 85 | 80 | 110 | 5340.71 | 3133215 | 14.84 | 0.81 | 0.27 | 335.97 | 182.95 | 5.47 | Seeder |
| Cha8 | Cha | mid | 100 | 90 | 130 | 7068.58 | 4900885 | 26.95 | 0.97 | 0.3 | 308.05 | 110.61 | 9.04 | Seeder |
| Cha9 | Cha | mid | 90 | 75 | 110 | 5301.44 | 3110177 | 15.22 | 0.87 | 0.34 | 385.32 | 220.76 | 4.53 | Seeder |
| Cha10 | Cha | mid | 85 | 70 | 100 | 4673.12 | 2492330 | 19.1 | 1.04 | 0.41 | 389.83 | 213.04 | 4.69 | Seeder |
| Csa6 | Csa | mid | 40 | 35 | 30 | 1099.56 | 175929.2 | 10.13 | 0.72 | 0.18 | 253.37 | 180.06 | 5.55 | Seeder |
| Csa7 | Csa | mid | 70 | 60 | 70 | 3298.67 | 1231504 | 15.93 | 1.33 | 0.39 | 293.48 | 244.38 | 4.09 | Seeder |
| Csa8 | Csa | mid | 70 | 65 | 55 | 3573.56 | 1048245 | 17.44 | 1.28 | 0.35 | 276.24 | 203.15 | 4.92 | Seeder |
| Csa9 | Csa | mid | 55 | 45 | 45 | 1943.86 | 466526.5 | 18.06 | 1.32 | 0.4 | 303.02 | 222.15 | 4.5 | Seeder |
| Csa10 | Csa | mid | 65 | 45 | 40 | 2297.29 | 490088.5 | 24.85 | 1.38 | 0.42 | 306.25 | 170.5 | 5.86 | Seeder |
| Sge6 | Sge | mid | 70 | 55 | 60 | 3023.78 | 967610.5 | 3.55 | 0.39 | 0.2 | 515.48 | 567.61 | 1.76 | Seeder |
| Sge7 | Sge | mid | 60 | 60 | 50 | 2827.43 | 753982.2 | 5.63 | 0.51 | 0.26 | 516.63 | 463.59 | 2.16 | Seeder |
| Sge8 | Sge | mid | 50 | 45 | 45 | 1767.15 | 424115 | 7.27 | 0.8 | 0.39 | 494.66 | 541.27 | 1.85 | Seeder |
| Sge9 | Sge | mid | 30 | 25 | 30 | 589.05 | 94247.78 | 3.4 | 0.28 | 0.15 | 551.72 | 447.06 | 2.24 | Seeder |
| Sge10 | Sge | mid | 80 | 70 | 80 | 4398.23 | 1876578 | 7.12 | 0.75 | 0.39 | 527.13 | 552.67 | 1.81 | Seeder |
| Cca6 | Cca | mid | 35 | 35 | 30 | 962.11 | 153938 | 3.05 | 0.15 | 0.05 | 352.43 | 173.44 | 5.77 | Seeder |
| Cca7 | Cca | mid | 30 | 20 | 25 | 471.24 | 62831.85 | 6.65 | 0.38 | 0.14 | 360.87 | 203.61 | 4.91 | Seeder |
| Cca8 | Cca | mid | 30 | 25 | 30 | 589.05 | 94247.78 | 3.54 | 0.19 | 0.07 | 380.17 | 202.54 | 4.94 | Seeder |
| Cca9 | Cca | mid | 35 | 30 | 35 | 824.67 | 153938 | 5.31 | 0.34 | 0.14 | 400.7 | 257.25 | 3.89 | Seeder |
| Cca10 | Cca | mid | 45 | 40 | 35 | 1413.72 | 263893.8 | 3.03 | 0.21 | 0.08 | 394.13 | 274.59 | 3.64 | Seeder |
| Ola6 | Ola | mid | 210 | 180 | 165 | 29688.05 | 26125485 | 15.75 | 1.28 | 0.53 | 411.88 | 334.03 | 2.99 | Resprouter |
| Ola7 | Ola | mid | 250 | 190 | 170 | 37306.41 | 33824481 | 13.88 | 0.87 | 0.3 | 343.99 | 214.7 | 4.66 | Resprouter |
| Ola8 | Ola | mid | 130 | 130 | 165 | 13273.23 | 11680441 | 18.88 | 1.21 | 0.44 | 368.34 | 235.38 | 4.25 | Resprouter |
| Ola9 | Ola | mid | 250 | 175 | 185 | 34361.17 | 33903021 | 13.58 | 0.89 | 0.36 | 409.55 | 268.56 | 3.72 | Resprouter |
| Ola10 | Ola | mid | 175 | 130 | 130 | 17867.81 | 12388347 | 20.4 | 1.19 | 0.45 | 378.65 | 220.1 | 4.54 | Resprouter |
| Cgr6 | Cgr | mid | 130 | 130 | 130 | 13273.23 | 9202772 | 6.4 | 0.92 | 0.37 | 407.66 | 585.31 | 1.71 | Seeder |
| Cgr7 | Cgr | mid | 100 | 95 | 95 | 7461.28 | 3780383 | 3.65 | 0.45 | 0.21 | 463.16 | 570.14 | 1.75 | Seeder |
| Cgr8 | Cgr | mid | 190 | 185 | 150 | 27606.75 | 22085396 | 5.84 | 0.75 | 0.29 | 388.84 | 498.97 | 2 | Seeder |
| Cgr9 | Cgr | mid | 90 | 90 | 120 | 6361.73 | 4071504 | 6.28 | 0.89 | 0.31 | 347.94 | 490.45 | 2.04 | Seeder |
| Cgr10 | Cgr | mid | 115 | 100 | 120 | 9032.08 | 5780530 | 6.7 | 0.79 | 0.29 | 365.13 | 431.34 | 2.32 | Seeder |
| Sro6 | Sro | mid | 85 | 75 | 110 | 5006.91 | 2937389 | 2.4 | 0.14 | 0.04 | 257.3 | 154.17 | 6.49 | Seeder |
| Sro7 | Sro | mid | 125 | 80 | 95 | 7853.98 | 3979351 | 4.48 | 0.28 | 0.08 | 288.34 | 182.14 | 5.49 | Seeder |
| Sro8 | Sro | mid | 100 | 65 | 80 | 5105.09 | 2178171 | 4.43 | 0.26 | 0.06 | 237.7 | 140.63 | 7.11 | Seeder |
| Sro9 | Sro | mid | 130 | 100 | 105 | 10210.18 | 5717699 | 5.42 | 0.4 | 0.13 | 313.07 | 232.84 | 4.29 | Seeder |
| Sro10 | Sro | mid | 75 | 70 | 120 | 4123.34 | 2638938 | 3.65 | 0.29 | 0.1 | 339.64 | 270.68 | 3.69 | Seeder |
| Cal11 | Cal | inland | 95 | 85 | 65 | 6342.09 | 2198591 | 2.11 | 0.2133 | 0.1042 | 488.5138 | 493.8389 | 2.024952 | Resprouter |
| Cal12 | Cal | inland | 55 | 50 | 55 | 2159.845 | 633554.5 | 3.27 | 0.3338 | 0.1713 | 513.1815 | 523.8532 | 1.908932 | Resprouter |
| Cal13 | Cal | inland | 70 | 60 | 60 | 3298.672 | 1055575 | 1.44 | 0.1662 | 0.0838 | 504.2118 | 581.9444 | 1.718377 | Resprouter |
| Cal14 | Cal | inland | 100 | 90 | 75 | 7068.583 | 2827433 | 2.57 | 0.327 | 0.1534 | 469.1131 | 596.8872 | 1.675359 | Resprouter |
| Cal15 | Cal | inland | 65 | 65 | 60 | 3318.307 | 1061858 | 3.59 | 0.2924 | 0.1668 | 570.4514 | 464.624 | 2.152278 | Resprouter |
| Cha11 | Cha | inland | 65 | 50 | 65 | 2552.544 | 884881.9 | 14.48 | 0.6685 | 0.2328 | 348.2423 | 160.7735 | 6.219931 | Seeder |
| Cha12 | Cha | inland | 40 | 30 | 50 | 942.4778 | 251327.4 | 10.9 | 0.4649 | 0.1837 | 395.1387 | 168.5321 | 5.933587 | Seeder |
| Cha13 | Cha | inland | 40 | 30 | 55 | 942.4778 | 276460.2 | 10.25 | 0.4982 | 0.2026 | 406.664 | 197.6585 | 5.05923 | Seeder |
| Cha14 | Cha | inland | 55 | 45 | 80 | 1943.86 | 829380.5 | 17.04 | 0.9205 | 0.3531 | 383.5959 | 207.2183 | 4.825828 | Seeder |
| Cha15 | Cha | inland | 45 | 40 | 50 | 1413.717 | 376991.1 | 14.49 | 0.6616 | 0.2596 | 392.3821 | 179.158 | 5.581664 | Seeder |
| Csa11 | Csa | inland | 60 | 40 | 55 | 1884.956 | 552920.3 | 21.35 | 1.0533 | 0.2886 | 273.996 | 135.1756 | 7.397782 | Seeder |
| Csa12 | Csa | inland | 50 | 45 | 55 | 1767.146 | 518362.8 | 25.38 | 1.2956 | 0.4208 | 324.7916 | 165.7998 | 6.031369 | Seeder |
| Csa13 | Csa | inland | 80 | 60 | 40 | 3769.911 | 804247.7 | 21.69 | 1.3874 | 0.3843 | 276.9929 | 177.1784 | 5.644028 | Seeder |
| Csa14 | Csa | inland | 45 | 40 | 45 | 1413.717 | 339292 | 29.65 | 2.123 | 0.7682 | 361.8464 | 259.0894 | 3.859672 | Seeder |
| Csa15 | Csa | inland | 85 | 50 | 55 | 3337.942 | 979129.7 | 23.78 | 1.4021 | 0.4023 | 286.9268 | 169.1758 | 5.911012 | Seeder |
| Sge11 | Sge | inland | 80 | 65 | 60 | 4084.07 | 1306903 | 4.48 | 0.5425 | 0.2806 | 517.235 | 626.3393 | 1.596579 | Seeder |
| Sge12 | Sge | inland | 80 | 60 | 60 | 3769.911 | 1206372 | 7.51 | 0.8036 | 0.4431 | 551.3937 | 590.0133 | 1.694877 | Seeder |
| Sge13 | Sge | inland | 115 | 80 | 65 | 7225.663 | 2504897 | 6.24 | 0.4202 | 0.2064 | 491.1947 | 330.7692 | 3.023256 | Seeder |
| Sge14 | Sge | inland | 60 | 45 | 45 | 2120.575 | 508938 | 7.24 | 0.6466 | 0.3442 | 532.3229 | 475.4144 | 2.103428 | Seeder |
| Sge15 | Sge | inland | 75 | 50 | 60 | 2945.243 | 942477.8 | 4.74 | 0.2928 | 0.1342 | 458.3333 | 283.1224 | 3.532042 | Seeder |
| Cca11 | Cca | inland | 35 | 25 | 25 | 687.2234 | 91629.79 | 3.49 | 0.2446 | 0.0827 | 338.103 | 236.9628 | 4.220073 | Seeder |
| Cca12 | Cca | inland | 45 | 40 | 45 | 1413.717 | 339292 | 6.14 | 0.3565 | 0.1494 | 419.0743 | 243.3225 | 4.109772 | Seeder |
| Cca13 | Cca | inland | 55 | 50 | 40 | 2159.845 | 460766.9 | 4.01 | 0.2565 | 0.0877 | 341.9103 | 218.7032 | 4.572406 | Seeder |
| Cca14 | Cca | inland | 60 | 40 | 40 | 1884.956 | 402123.9 | 3.26 | 0.2161 | 0.0755 | 349.3753 | 231.5951 | 4.317881 | Seeder |
| Cca15 | Cca | inland | 50 | 40 | 40 | 1570.796 | 335103.2 | 2.46 | 0.1635 | 0.056 | 342.5076 | 227.6423 | 4.392857 | Seeder |
| Ola11 | Ola | inland | 260 | 230 | 155 | 46966.81 | 38825896 | 13.08 | 0.771 | 0.3185 | 413.0999 | 243.5015 | 4.10675 | Resprouter |
| Ola12 | Ola | inland | 230 | 210 | 180 | 37934.73 | 36417342 | 11.3 | 0.6122 | 0.2322 | 379.2878 | 205.4867 | 4.866494 | Resprouter |
| Ola13 | Ola | inland | 165 | 165 | 115 | 21382.47 | 13114579 | 12.05 | 0.8354 | 0.3533 | 422.9112 | 293.195 | 3.410699 | Resprouter |
| Ola14 | Ola | inland | 180 | 125 | 120 | 17671.46 | 11309734 | 15.53 | 1.0437 | 0.4372 | 418.8943 | 281.5196 | 3.55215 | Resprouter |
| Ola15 | Ola | inland | 105 | 95 | 80 | 7834.347 | 3342655 | 26 | 1.7094 | 0.803 | 469.7555 | 308.8462 | 3.237858 | Resprouter |
| Cgr11 | Cgr | inland | 140 | 140 | 95 | 15393.8 | 7799527 | 6.34 | 0.8611 | 0.3821 | 443.7348 | 602.6814 | 1.659252 | Seeder |
| Cgr12 | Cgr | inland | 105 | 100 | 95 | 8246.681 | 4178318 | 5.15 | 0.5795 | 0.2131 | 367.7308 | 413.7864 | 2.416706 | Seeder |
| Cgr13 | Cgr | inland | 120 | 80 | 120 | 7539.822 | 4825486 | 4.68 | 0.6607 | 0.2663 | 403.0574 | 569.0171 | 1.757416 | Seeder |
| Cgr14 | Cgr | inland | 90 | 90 | 130 | 6361.725 | 4410796 | 6.01 | 0.8208 | 0.3209 | 390.96 | 533.9434 | 1.872858 | Seeder |
| Cgr15 | Cgr | inland | 120 | 120 | 105 | 11309.73 | 6333451 | 5.94 | 0.6532 | 0.2611 | 399.7244 | 439.5623 | 2.27499 | Seeder |
| Sro11 | Sro | inland | 40 | 35 | 80 | 1099.557 | 469144.5 | 4.32 | 0.2671 | 0.0577 | 216.024 | 133.5648 | 7.487002 | Seeder |
| Sro12 | Sro | inland | 65 | 115 | 90 | 5870.851 | 2818009 | 3.9 | 0.2207 | 0.0486 | 220.2084 | 124.6154 | 8.024691 | Seeder |
| Sro13 | Sro | inland | 70 | 50 | 95 | 2748.894 | 1392773 | 3.61 | 0.1721 | 0.0361 | 209.7618 | 100 | 10 | Seeder |
| Sro14 | Sro | inland | 35 | 30 | 55 | 824.6681 | 241902.6 | 4.05 | 0.2636 | 0.0696 | 264.0364 | 171.8519 | 5.818966 | Seeder |
| Sro15 | Sro | inland | 35 | 25 | 45 | 687.2234 | 164933.6 | 2.37 | 0.1506 | 0.0533 | 353.9177 | 224.8945 | 4.446529 | Seeder |

**Table S2.** Significant squared correlation coefficients (r²) between functional traits and the PCA axes, derived from the PCA conducted to characterize the community's main functional trends, and the NMS ordination

| **Variables** | **r^2^** |
| --- | --- |
| Plant height | 0.27*** |
| Plant area | 0.32*** |
| Plant volume | 0.10** |
| Leaf Area | 0.69*** |
| LMA | 0.55*** |
| SLA | 0.64*** |
| Seeder cover | 0.02 |
| Resprouter cover | 0.02 |
| PC1 | 0.64*** |
| PC2 | 0.18*** |

**p<0.01 and ***p<0.001.

**Table S3.** Results of the Moran’s I test for spatial autocorrelation of woody plant species along three transects (coastal, mid, and inland) sampled in 2021 and 2022. The analysis is based on a one-dimensional neighbourhood structure (adjacent sampling points). The “Observed” column shows the Moran’s I statistic for each species, indicating spatial clustering (positive values), dispersion (negative values), or randomness (values near zero). The “Expected” value corresponds to the Moran’s I under a null model of spatial randomness.

| **Year** | **Zone** | **Species** | **Observed** | **Expected** | **p-value** |  |
| --- | --- | --- | --- | --- | --- | --- |
| 2021 | coastal | Cal | -0,0328779 | -0,0526316 | 0,4653747 |  |
| 2021 | coastal | Cha | 0,4018265 | -0,0526316 | 0,0185141 | * |
| 2021 | coastal | Cca | 0,3301618 | -0,0526316 | 0,037638 | * |
| 2021 | coastal | Csa | -0,25 | -0,0526316 | 0,848762 |  |
| 2021 | coastal | Cyt | -0,0526316 | -0,0526316 | 0,5 |  |
| 2021 | coastal | Sge | 0,1196319 | -0,0526316 | 0,2061932 |  |
| 2021 | coastal | Jox | -0,0526316 | -0,0526316 | 0,5 |  |
| 2021 | mid | Cal | -0,2757318 | -0,0526316 | 0,8843605 |  |
| 2021 | mid | Cha | -0,1581921 | -0,0526316 | 0,6791121 |  |
| 2021 | mid | Cca | 0,1150442 | -0,0526316 | 0,2233492 |  |
| 2021 | mid | Csa | -0,016129 | -0,0526316 | 0,4347285 |  |
| 2021 | mid | Cli | -0,0263158 | -0,0526316 | 0,0126737 | * |
| 2021 | mid | Ola | 0,0684932 | -0,0526316 | 0,2872558 |  |
| 2021 | mid | Cyt | 0,219348 | -0,0526316 | 0,0873171 | . |
| 2021 | mid | Sge | 0,0782443 | -0,0526316 | 0,2796802 |  |
| 2021 | mid | Jox | -0,0526316 | -0,0526316 | 0,5 |  |
| 2021 | mid | Sro | -0,1512346 | -0,0526316 | 0,6743218 |  |
| 2021 | mid | Hpi | -0,0714286 | -0,0526316 | 0,5909032 |  |
| 2021 | inland | Cal | 0,3342607 | -0,0526316 | 0,0372915 | * |
| 2021 | inland | Cha | -0,0070093 | -0,0526316 | 0,4119386 |  |
| 2021 | inland | Cca | -0,1598361 | -0,0526316 | 0,6822346 |  |
| 2021 | inland | Csa | 0,1424978 | -0,0526316 | 0,1845753 |  |
| 2021 | inland | Cli | -0,0869565 | -0,0526316 | 0,6286444 |  |
| 2021 | inland | Ola | -0,1226415 | -0,0526316 | 0,6775188 |  |
| 2021 | inland | Cyt | 0,2611301 | -0,0526316 | 0,0804575 | . |
| 2021 | inland | Sge | 0,1240267 | -0,0526316 | 0,2179877 |  |
| 2021 | inland | Sro | 0,3625954 | -0,0526316 | 0,0245479 | * |
| 2022 | coastal | Cal | -0,0127796 | -0,0526316 | 0,4245954 |  |
| 2022 | coastal | Cha | -0,2137203 | -0,0526316 | 0,8321972 |  |
| 2022 | coastal | Cca | 0,5481481 | -0,0526316 | 0,0020993 | ** |
| 2022 | coastal | Csa | -0,1298701 | -0,0526316 | 0,7110551 |  |
| 2022 | coastal | Cyt | 0,3076923 | -0,0526316 | 0,0051785 | ** |
| 2022 | coastal | Sge | 0,0384968 | -0,0526316 | 0,3371745 |  |
| 2022 | coastal | Sro | -0,0526316 | -0,0526316 | 0,5 |  |
| 2022 | coastal | Hpi | -0,0526316 | -0,0526316 | 0,5 |  |
| 2022 | mid | Cal | 0,1120401 | -0,0526316 | 0,2162166 |  |
| 2022 | mid | Cha | -0,125 | -0,0526316 | 0,6277655 |  |
| 2022 | mid | Cca | -0,2758319 | -0,0526316 | 0,8436446 |  |
| 2022 | mid | Csa | -0,089315 | -0,0526316 | 0,5725132 |  |
| 2022 | mid | Cli | -0,0526316 | -0,0526316 | 0,5 |  |
| 2022 | mid | Ola | -0,0491071 | -0,0526316 | 0,4923101 |  |
| 2022 | mid | Cyt | 0,0397165 | -0,0526316 | 0,3337922 |  |
| 2022 | mid | Sge | 0,0216638 | -0,0526316 | 0,3663985 |  |
| 2022 | mid | Sro | 0,25 | -0,0526316 | 0,0670491 | . |
| 2022 | inland | Cal | -0,073991 | -0,0526316 | 0,5384894 |  |
| 2022 | inland | Cha | -0,0426313 | -0,0526316 | 0,4818967 |  |
| 2022 | inland | Cca | 0,2147766 | -0,0526316 | 0,1216228 |  |
| 2022 | inland | Csa | 0,247331 | -0,0526316 | 0,0827555 | . |
| 2022 | inland | Cli | 0,0967742 | -0,0526316 | 0,2185042 |  |
| 2022 | inland | Ola | 0,2413793 | -0,0526316 | 0,0704115 | . |
| 2022 | inland | Cyt | -0,1289986 | -0,0526316 | 0,6450463 |  |
| 2022 | inland | Sge | 0 | -0,0526316 | 0,4059058 |  |
| 2022 | inland | Sro | 0,2748447 | -0,0526316 | 0,0382675 | * |

**Table S4.** Moran’s I statistics for the spatial autocorrelation of total woody plant abundance along the three linear transects (coastal, mid, and inland) in 2021 and 2022. The analysis is based on a one-dimensional neighbourhood structure (adjacent sampling points). Positive values indicate spatial clustering of woody individuals, negative values suggest spatial dispersion, and values near zero indicate random spatial distribution. The “Expected” value represents the Moran’s I expected under a null model of spatial randomness.

| **Year** | **Zone** | **Observed** | **Expected** | **p-value** |  |
| --- | --- | --- | --- | --- | --- |
| 2021 | coastal | -0,221637 | -0,052632 | 0,7826388 |  |
| 2021 | mid | 0,2148639 | -0,052632 | 0,1170255 |  |
| 2021 | inland | 0,0381862 | -0,052632 | 0,3377979 |  |
| 2022 | coastal | -0,198528 | -0,052632 | 0,7450125 |  |
| 2022 | mid | 0,2673774 | -0,052632 | 0,0634554 | . |
| 2022 | inland | -0,113091 | -0,052632 | 0,6090395 |  |

**Table S5.** Moran’s I values assessing the spatial autocorrelation of total woody plant abundance along each transect (coastal, mid, inland) in 2021 and 2022. The “Observed” column shows the Moran’s I statistic calculated spatial data considering the distance between transects. The “Expected” value corresponds to Moran’s I under the null hypothesis of spatial randomness.

| **Year** | **Zone** | **Observed** | **Expected** | **p-value** |  |
| --- | --- | --- | --- | --- | --- |
| 2021 | coastal | -0,246199 | -0,052632 | 0,8276262 |  |
| 2021 | mid | 0,2185431 | -0,052632 | 0,1013284 |  |
| 2021 | inland | 0,0423628 | -0,052632 | 0,321943 |  |
| 2022 | coastal | -0,228484 | -0,052632 | 0,7991821 |  |
| 2022 | mid | 0,1701884 | -0,052632 | 0,130845 |  |
| 2022 | inland | -0,105401 | -0,052632 | 0,6006999 |  |

**Figure S1.** Spatial distribution of woody plant abundance along three transects (Coast, Mid, and Interior) sampled in 2021 and 2022 using the point–intercept method. Each point represents a sampling location along a 200-meter transect (20 points per transect), with point size proportional to total woody plant abundance. Colours represent different transects: blue for Coastal, teal for Mid, and green for Interior.


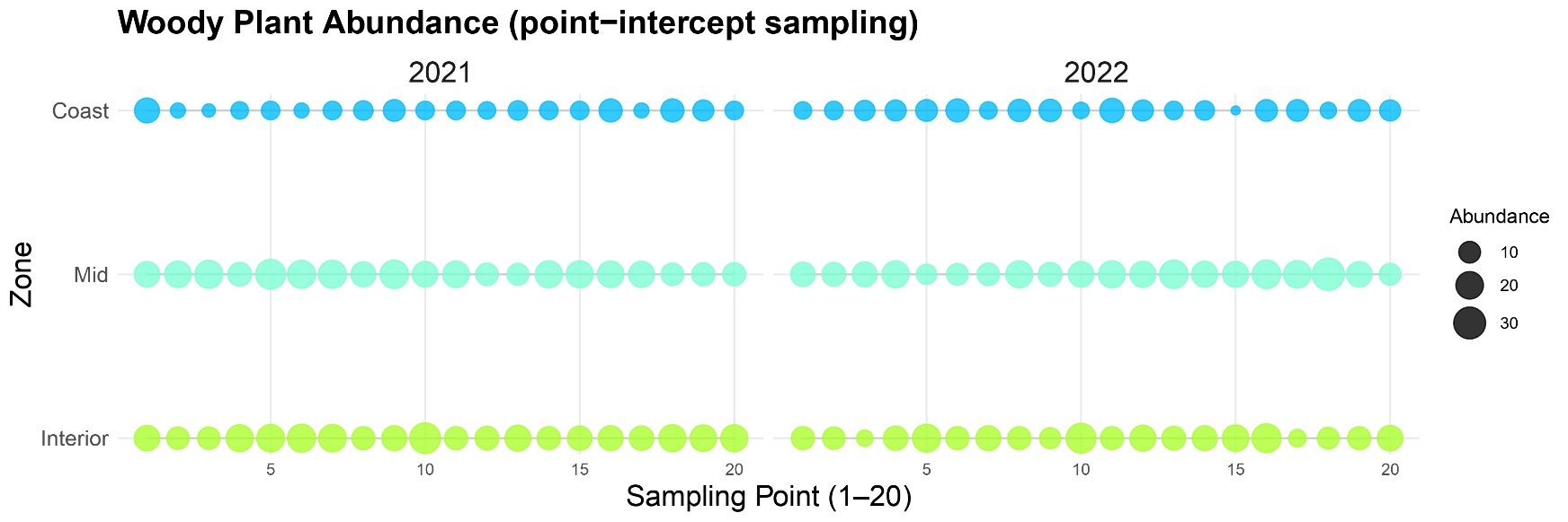

Supplement: Supplementary file 1 — Supplementary Material 1. [file 41598_2025_4400_MOESM1_ESM.docx]
